# Impact of meningioma surgery on use of antiepileptic, antidepressant, and sedative drugs: A Swedish nationwide matched cohort study

**DOI:** 10.1002/cam4.3868

**Published:** 2021-03-26

**Authors:** Erik Thurin, Isabelle Rydén, Thomas Skoglund, Anja Smits, Sasha Gulati, Göran Hesselager, Jiri Bartek, Roger Henriksson, Øyvind Salvesen, Asgeir S. Jakola

**Affiliations:** ^1^ Institute of Neuroscience and Physiology University of Gothenburg Sahlgrenska Academy Gothenburg Sweden; ^2^ Department of Neurosurgery Sahlgrenska University Hospital Gothenburg Sweden; ^3^ Department of Neurosurgery St.Olavs University Hospital HF Trondheim Norway; ^4^ Institute of Neuroscience Norwegian University of Science and Technology Trondheim Norway; ^5^ Department of Neurosurgery Uppsala University Hospital Uppsala Sweden; ^6^ Department of Neurosurgery Karolinska University Hospital Stockholm Sweden; ^7^ Department of Clinical Neuroscience and Department of Medicine Karolinska Institutet Stockholm Sweden; ^8^ Department of Neurosurgery Rigshospitalet Copenhagen Denmark; ^9^ Department of Radiation Sciences & Oncology University of Umeå Umea Sweden; ^10^ Department of Public Health and Nursing Norwegian University of Science and Technology Trondheim Norway

**Keywords:** antidepressant, anti‐epileptic drugs, neurosurgery, primary brain tumor, quality of life

## Abstract

**Background:**

Meningioma is the most common primary intracranial tumor and surgery is the main treatment modality. As death from lack of tumor control is rare, other outcome measures like anxiety, depression and post‐operative epilepsy are becoming increasingly relevant. In this nationwide registry‐based study we aimed to describe the use of antiepileptic drugs (AED), antidepressants and sedatives before and after surgical treatment of an intracranial meningioma compared to a control population, and to provide predictors for continued use of each drug‐group two years after surgery.

**Methods:**

All adult patients with histopathologically verified intracranial meningiomas were identified in the Swedish Brain Tumor Registry and their data were linked to relevant national registries after assigning five matched controls to each patient. We analyzed the prescription patterns of antiepileptic drugs (AED), antidepressants and sedative drugs in the two years before and the two years following surgery.

**Results:**

For the 2070 patients and 10312 controls identified the use of AED, antidepressants and sedatives was comparable two years before surgery. AED use at time of surgery was higher for patients than for controls (22.2% vs. 1.9%, *p* < 0.01), as was antidepressant use (12.9% vs. 9.4%, *p* < 0.01). Both AED and antidepressant use remained elevated after surgery, with patients having a higher AED use (19.7% vs. 2.3%, *p* < 0.01) and antidepressant use (14.8% vs. 10.6%, *p* < 0.01) at 2 years post‐surgery. Use of sedatives peaked for patients at the time of surgery (14.4% vs. 6.1%, *p* < 0.01) and remained elevated at two years after surgery with 9.9% versus 6.6% (*p* < 0.01). For all the studied drugs, previous drug use was the strongest predictor for use 2 years after surgery.

**Conclusion:**

This nationwide study shows that increased use of AED, antidepressants and sedatives in patients with meningioma started perioperatively, and remained elevated two years following surgery.

## Graphical abstract

By coupling a nationwide cancer registry of 2070 patients with data on purchased drugs and diagnoses, it was shown that after meningioma surgery the use of antiepileptics, antidepressants and sedatives was distinctly increased compared to controls. As this may be an often‐overlooked factor in deciding whether to perform surgery, predictors for identifying especially vulnerable patients are explored.

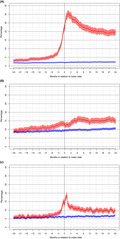

## 1 INTRODUCTION

Meningiomas are the most common primary intracranial tumors.^1^, ^2^, ^3^ Surgical treatment has been shown to increase survival^4^ and quality of life,^5^ while more extensive resection is related to a lower recurrence rate.^6^ However, as complications and neurological deterioration related to tumor resection are not trivial,^2^, ^7^, ^8^ careful risk‐benefit considerations are essential for optimal management.

Epilepsy is a common presenting symptom of meningioma,^8^ and seizure control is an important outcome measure after surgery.^9^ While patients may experience seizure remission after meningioma surgery, the reported chance for this varies widely (38%–72%) between studies. ^9^, ^10^, ^11^, ^12^ There is also a considerable risk of new onset seizures in previously seizure free patients, varying between 6 and 19% between studies. ^9^, ^10^, ^12^, ^13^, ^14^, ^15^, ^16^, ^17^, ^18^ Such a cross‐over of symptoms is likely to mask both the positive and negative impacts of surgery. However, interpretation of available studies is difficult as the time‐point of follow‐up varies considerably between studies and is sometimes vaguely defined. To better understand these cross‐over events, we need to study the exact time‐patterns with a high resolution.

It is unclear if patients undergoing meningioma surgery have an increased rate of depression preoperatively. While preoperative depression seems related to impaired outcome,^19^ surgery may^20^ or may not^21^ improve depressive symptoms. Also, patients treated with psychotropic medication could undergo a disproportionate rate of brain imaging, with increased risk of incidental meningioma diagnosis and possible overtreatment.^22^, ^23^ Even less is known about use of sedative drugs in patients with meningioma.

In this nationwide registry‐based study we aimed to describe the use of AED, antidepressants and sedatives before and after surgical treatment of an intracranial meningioma compared to a control population, and to provide predictors for continued use of each drug‐group two years after surgery.

## 2 MATERIALS AND METHODS

We combined data from several nationwide Swedish registries, linked through the unique personal identification numbers given to all Swedish citizens. The methods have been described in detail previously,^24^ but the procedure is described in short below.

### 2.1 Linking of registries

The Swedish Brain Tumor Registry (SBTR) is a nationwide registry of adult (≥18 years) patients with primary brain tumors, with a surgically acquired pathohistological diagnosis. In SBTR we identified all patients with a first‐time histological diagnosis of intracranial meningioma according to the 2007 WHO classification of brain tumors, and a day of surgery/index date between April 1^st^ 2009 and December 31^st^ 2015.^25^ Patients with radiologically suspected meningioma without histological diagnosis were not included in the present study. As a criterion to be included the annual rate of registration per region needed to be above 80% as compared to the compulsory National Cancer Registry. This was done to provide population‐based data. Therefore, for one region, we included data only from January 1^st^ 2012 to December 31^st^ 2013 while for all other regions patients were included for the entire study period.

**FIGURE 1:**
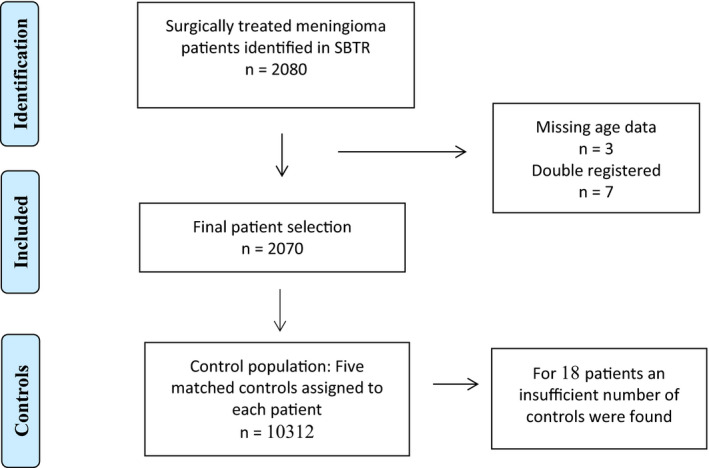
Flow chart of patient selection

Statistics Sweden (www.scb.se) provided data on educational level and income for the patients included in the analyses. Furthermore, a control population of five individuals for each patient was obtained from the general population. The control population was matched with respect to year of birth, sex, municipality of residence and educational level. For 18 patients the number of controls per patient was incomplete. This rendered a patient population of 2070 and a control population of 10312 individuals. A flow‐chart of the inclusion and exclusion of patients and controls is available in Figure  1.

From the Swedish National Board of Health and Welfare we received data from the *National Patient Registry* (NPR) and the *National Prescription Registry* (NPrP). The NPR included information on ICD‐code and date of inpatient and outpatient visits, including diagnostic and procedural codes, in the period 2007–2016. It included private and public hospitals, but no primary care contacts. The ICD‐10 codes were used to classify comorbidity according to the Elixhauser comorbidity index.^26^, ^27^ The prescription registry provided information on date of dispensing and type of drug according to the Anatomical Therapeutic Chemical (ATC) classification system, in the period from 2007 through 2017. Data on drug use were based on purchase of prescription drugs by the patient, please see Table 1 for ATC‐based definitions of drug groups. Active use of a prescription group was defined as having purchased a drug of this prescription group in the prior 90 days. For sedatives, the patient was considered an active user only for 30 days after a purchase since sedatives are more often used as a short‐term drug. When calculating active users as percentage of the population, only living individuals were considered. The registries under The National Board of Health and Welfare were accessed on March 16^th^ 2018.

**TABLE 1:** Definition of variables, and the source of data used for calculating each variable

Variable	Definition	Source of data
Index date	Date of surgery for patients. Controls received the same index date as their respective cases.	SBTR
Radiotherapy	Registered as “yes” if indicated in SBTR and/or if procedure codes in NPR indicated administration of radiotherapy.	SBTR
Elixhauser comorbidity index	According to index. The conditions removed from the index due to possible association with diagnosis of meningioma were: G40 Epilepsy, G41 Status epilepticus, R56 Convulsions, R47 Dysphasia/aphasia, C70‐72: Malignant tumor in central nervous system. With this index both cases and controls were provided with a score from 0–30 based upon comorbid categories present or not. We report as 0, 1, 2, ≥3 categories present. The ICD−10 data used to classify comorbidity were from NPR the 2 years prior to index date	NPR
Prescription group	All drugs with a common ATC‐code. Groups were defined as follows: **Antiepileptics (AED):** ATC class N03A (antiepileptics), except N03AX12 (Gabapentin) and N03AX16 (Pregabalin) **Antidepressants:** ATC class N06A (antidepressants) **Sedatives:** ATC class N05B (anxiolytics, including benzodiazepines) and N05C (hypnotics and sedatives)	NPrR
Active use	Active use of a prescription group was defined as having purchased a drug of this prescription group in the prior 90 days. For the prescription group “Sedatives” the patient was considered an active user only for 30 days after a purchase. When calculating the percentage of the population that is active users, only alive individuals were considered.	NPrR

### 2.2 Statistics

Data from the registries were imported into corresponding tables in a mySQL database. Data on purchased prescription drugs use were individually analyzed for each patient (date and ATC‐code) and each control and was then combined with clinical data using Python as described earlier.^28^ Definitions regarding index date, active use, and prescription groups are provided in Table 1.

Other data derivations were done using mySQL. R version 2.13.1 was used for statisticasl analyses. For each day, from two years (730 days) prior through two years after index date, the percentage of all alive patients and controls that were users of the defined prescription groups was analyzed. Continuous variables were summarized using the median, first and third quartiles and compared between cases and controls using the Mann‐Whitney U test. Categorical variables were summarized using counts and proportions and compared between cases and controls using the Fisher's exact test.

To identify relevant predictors for use of AED, antidepressants and sedatives at end of follow‐up, we performed multivariable regression analyses, see Table S1–S3. Covariates were chosen based upon presumed relevance.

## 3 RESULTS

### 3.1 Demographic data

Baseline characteristics of the 2070 patients included in this study are presented in Table 2. A comparison between patients and controls regarding socioeconomic variables and comorbidities is presented in Table 3.

**TABLE 2:** Baseline and treatment characteristics for patients with meningioma. (n = 2070)

Variable	Meningioma patients (n = 2070)
Age, median (First quartile(Q1), Third quartile(Q3))	60 (49, 69)
Female, n (%)	1449, (70.0)
Asymptomatic, n (%)	294 (14.2)
WHO functional status, n (%)	
0, fully active	933 (45.1)
1, light work possible	566 (27.3)
2, cares for self	305 (14.7)
3, limited self care	144 (7.0)
4, disabled, confined to bed	20 (1.0)
Missing	102
Tumor laterality, n (%)	
Left	736 (35.6)
Right	856 (41.4)
Bilateral	123 (5.9)
Missing	355
Localization, n (%)	
Skull‐base	290 (14.0)
Not Skull‐base	1780 (86.0)
Tumor size, n (%)	
<4 cm	924 (52.8)
4–6 cm	597 (34.1)
>6 cm	230 (13.1)
Missing	319
Simpson grade, n (%)	
Grade I‐III	1587 (86.5)
Grade IV‐V	247 (13.5)
Missing	236
New deficit after surgery, n (%)	310 (15.0)
Missing	4
Reoperation due to complication, n (%)	103 (5.0)
Missing	3
WHO grade, n (%)	
Grade I	1803 (87.1)
Grade II‐III	267 (12.9)
Oncological treatment planned, n (%)	120 (5.9)
Missing	27
Wait time from radiological diagnosis (surgery date‐radiological diagnosis), weeks median (Q1,Q3)	10 (4, 24)

**TABLE 3:** Socioeconomic variables and comorbidities of patients and controls

	Meningioma n = 2070	Controls, n = 10,312	*p*‐value
Educational level, at index year, n (%)			
Basic to high‐school	1375 (69.2)	7019 (69.2)	0.98
Higher education	612 (30.8)	3131 (30.8)	
Missing	83	162	
Disposable income, n (%)	180 k (132 k,	199 k (137 k,	<0.001
Median (Q1, Q3)	256 k)	277 k)	
Elixhauser comorbidities at index date, n (%)			
0	1067 (51.5)	7126 (69.1)	<0.001
1	523 (25.3)	1671 (16.2)	
2	254 (12.3)	754 (7.3)	
3 or more	226 (10.9)	761 (7.4)	

### 3.2 Drug use patterns

**FIGURE 2:**
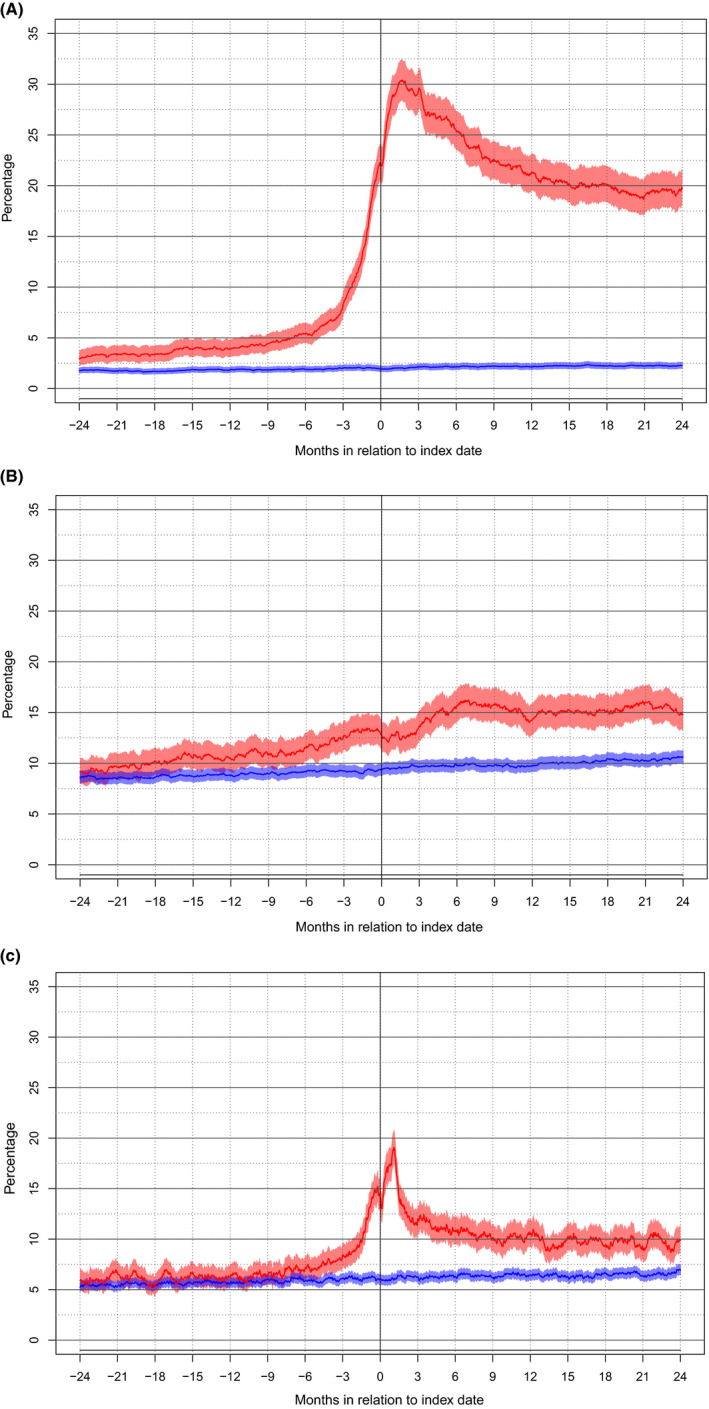
(A‐C) Graph representing the proportions (95% CI) of patients (red) and controls (blue) with active use of the following prescription groups two years prior to index date through two years following index date: (A) AED, (B) Antidepressants, (C) Sedatives

The percentage of patients and controls with an active use of each of the defined prescription groups are presented for the index date and 2 years after surgery in Table 4, and for the entire time interval from two years before until two years following index date in Figure 2A‐C.

**TABLE 4:** Drug use of patients and controls

	Meningioma n = 2070		Controls, n = 10312		*p*‐value
	n (%)	95% CI	n (%)	95% CI	
Use of AED at index date, n (%)	455 (22.0)	20.2–23.8	200 (1.9)	1.7–2.2	<0.001
Use of antidepressants at index date, n (%)	266 (12.9)	11.4–14.4	968 (9.4)	8.8–10.0	<0.001
Use of sedatives at index date, n (%)	298 (14.4)	12.9–16.0	627 (6.1)	5.6–6.6	<0.001
Alive at 2 years after index date, n (%)	1957 (94.5)	93.5–95.5	10082 (97.8)	97.5–98.0	<0.001
AED use at 2 years after index date, n (% of alive)	386 (19.7)	18.0–21.6	231 (2.3)	2.0–2.6	<0.001
Use of antidepressants at 2 years after index date, n (% of alive)	289 (14.8)	13.2–16.4	1072 (10.6)	10.0–11.3	<0.001
Sedatives use at 2 years after index date, n (% of alive)	193 (9.9)	8.6–11.3	698 (6.9)	6.4–7.4	<0.001

As seen in Figure 2A the rate of AED use for patients at the date of surgery was 22.2% compared to 1.9% for controls (*p* < 0.01). The rate for patients remained high with 18.6% of patients and 2.2% of controls using AED at two years after index date (*p* < 0.01). As seen in Figure 2B, the corresponding rate of antidepressant use was 12.9% for patients and 9.4% of controls at index date (*p* < 0.01). However, while AED use decreased over time, antidepressant use increased during follow‐up for both patients with 14.0% use at two years after index date and controls with 10.4% use at two years after index date (*p* < 0.01). As seen in Figure 2C the use of sedatives was elevated for patients compared to controls with 14.2% versus 6.0% use at index date (*p* < 0.01) and remained somewhat elevated at two years after surgery with 9.9% versus 6.6% (*p* < 0.01). Concerning symptom cross‐over (e.g. seizure remission and induced seizures) a summary of changes in use of AED, antidepressants and sedatives at index date and at two years after surgery is provided in Tables 5, 6, 7.

**TABLE 5:** Pattern of change in AED treatment, for patients and controls

Group (n)	Use of AED at index date	N (% of group)	Use of AED at 2‐year follow‐up	N (% of subgroup)
Patients (2070)	Yes	455 (22.0)	Yes	219 (48.1)
			No	219 (48.1) *
			Dead	17 (3.7)
	No	1615 (78.0)	Yes	167 (10.3)^b^
			No	1352 (83.7)
			Dead	96 (5.9)
Controls (10,312)	Yes	200 (1.9)	Yes	137 (68.5)
			No	54 (27.0)^a^
			Dead	9 (4.5)
	No	10112 (98.1)	Yes	94 (0.9)^b^
			No	9797 (96.9)
			Dead	221 (2.2)

^a^ Use of AED at index date but no use of AED at 2‐year follow‐up was 48.1% for patients and 27.0% for controls (*p* < 0.001).

^b^ Use of AED at 2‐year follow‐up but not at index date occurred for 10.3% of patients and 0.9% of controls (*p* < 0.001).

**TABLE 6:** Pattern of change in antidepressant treatment, for patients and controls

Group (n)	Use of antidepressant at index date	N (% of group)	Use of antidepressant at 2‐year follow‐up	N (% of subgroup)
Patients (2070)	Yes	264 (12.8%)	Yes	133 (50.4%)
			No	113 (42.8%)^a^
			Dead	18 (6.8%)
	No	1806 (87.2%)	Yes	156 (8.6%)^b^
			No	1555 (86.1%)
			Dead	95 (5.3%)
Controls (10,312)	Yes	963 (9.3%)	Yes	647 (67.2%)
			No	266 (27.6%)^a^
			Dead	50 (5.2%)
	No	9349 (90.7%)	Yes	425 (4.5%)^b^
			No	8744 (93.6%)
			Dead	180 (1.9%)

^a^ Use of antidepressants at index date but no use of antidepressants at 2‐year follow‐up was 42.8% for patients and 27.6% for controls (*p* < 0.001).

^b^ Use of antidepressants at 2‐year follow‐up but not at index date occurred for 8.6% of patients and 4.5% of controls (*p* < 0.001).

**TABLE 7:** Pattern of change in sedative treatment, for patients and controls

Group (n)	Use of sedatives at index date	N (% of group)	Use of sedatives at 2‐years after index date	N (% of subgroup)
Patients (2070)	Yes	298 (14.4)	Yes	87 (29.2)
			No	188 (62.6)^a^
			Dead	23 (7.8)
	No	1772 (85.6)	Yes	106 (6.0)^b^
			No	1576 (88.9)
			Dead	90 (5.1)
Controls (10,312)	Yes	627 (6.1)	Yes	309 (49.3)
			No	278 (44.3)^a^
			Dead	40 (6.4)
	No	9685 (93.9)	Yes	389 (4.0)^b^
			No	9106 (94.0)
			Dead	190 (2.0)

^a^ Use of sedatives at index date but no use of sedatives at 2‐year follow‐up was 62.6% for patients and 44.3% for controls (*p* < 0.001).

^b^ Use of sedatives at 2‐year follow‐up but not at index date occurred for 6.0% of patients and 4.0% of controls (*p* < 0.001).

### 3.3 Predictors of drug use

A logistic regression model was created to identify potential predictors for active use of AED at the end of follow‐up (Table S1). Previous AED use, more comorbidities, a new neurological deficit after surgery, larger tumor size, worse WHO functional status, and planned postoperative oncological treatment (i.e. radiation) were associated with an increased risk of being on active use of AED at two years after surgery. Higher level of education and longer wait time from radiological diagnosis to surgery decreased the risk of AED use at two years after surgery.

For antidepressants, use at two years after surgery was predicted by previous use of antidepressants and worse WHO functional status (Table S2). For sedatives, use at two years after surgery was predicted by previous sedative use, higher age, more comorbidities, a new neurological deficit after surgery and worse WHO functional status (Table S3).

## 4 DISCUSSION

This nationwide registry‐based study demonstrates that use of AED, antidepressant drugs and to a lesser degree also sedative drugs, is elevated in patients with meningioma compared to a matched control group, both at the time of surgery and two years after. Furthermore, there was no favorable trend postoperatively with less use of AED, antidepressant drugs or sedative drugs as compared to preoperative use, although a substantial shift in AED and antidepressant users was seen.

### 4.1 Antiepileptics

There was a marked increase in the use of AED, starting approximately six months prior to surgery, whereas controls had stable AED use (around 2%) for the entire studied period. A previous meta‐analysis based on 4709 patients reported a mean preoperative seizure rate of 29%.^17^ This is in line to our finding of 22% of patients with AED use at index date. The two year rate of AED use in our study of 19.7% is comparable to several studies reporting post‐operative seizure rates of 15%–30%.^9^, ^10^, ^12^, ^29^, ^30^

The rate of new‐onset AED use in previously AED naïve patients was 10% in our study, and in line with several previous studies reporting new onset seizure rates of 6%–19%.^9^, ^10^, ^12^, ^13^, ^14^, ^15^, ^16^, ^17^ Interestingly, this does not seem to have improved much with microsurgical techniques as historical case series indicate a similar rate (6%) as far back as 1935.^12^ It is also important to determine to what extent patients with seizures become seizure free after surgery. There is considerable variability in the degree of seizure relief that is reported after meningioma surgery (38%–72%) between different studies,^9^, ^10^, ^11^, ^12^ In our material, 52% of the patients using AED at the date of surgery were no longer active users at the end of our two‐year follow up. This figure is somewhat lower than the rate of seizure relief of 69% reported by the above mentioned meta‐analysis.^17^ It is worth noting however that a higher pre‐operative seizure rate was also reported and they registered seizure freedom rather than AED use. Some patients in our material with AED use before surgery, on the other hand, may have continued active use of AED for the entire two‐year follow‐up period without having any seizure during this time. Thus, the difference is probably to some extent related to a different study design and differences in definition, for example, if a seizure during the first 7 days should be included. Also of note, primary prevention with AED is generally avoided in Sweden for patients with primary brain tumors. Differences in guidelines concerning preventive AED could affect the reported postoperative use of AED and seizure rate, but such primary prophylaxis is generally considered not to affect the long‐term development of epilepsy.^31^

Identifying risk factors for postoperative seizures is of particular interest for daily clinical practice, as there are indications that AED use among patients with meningioma has a negative impact on quality of life.^32^ STAMPE2 is a prognostic index developed to predict the risk of postoperative seizures after meningioma surgery.^9^ Our results only partially concur with the STAMPE2 scoring system for predicting postoperative seizures. New onset neurological deficits and preoperative epilepsy were useful predictors in both STAMPE2 and our study. However, we were surprised to find that age and reoperation due to complication were not significant predictors in our model. However, according to our model it seems like complex cases in terms of more comorbidity, lower functional status and larger tumors have an increased risk of postoperative AED use.

### 4.2 Antidepressants

A prescription of an antidepressant is an indicator that a patient has some form of mental distress, the main indication is depression.^33^ In patients with meningioma preoperative depression has been negatively associated to clinical outcome, including survival.^19^ In our study, the use of antidepressants was not different between cases and controls at two years before surgery but was significantly elevated from around one year prior to surgery. Since the increase in use of antidepressants preceded the median wait time from diagnosis to surgery, it seems unlikely that the only cause of the increase in antidepressant use is the psychological reaction to the meningioma diagnosis. It can, conversely, be speculated that patients with depression are more likely to receive a CT or MRI of the brain, increasing the probability of discovering an otherwise asymptomatic meningioma. In line with this notion, short‐term antidepressant use is associated with an increased risk of being diagnosed with meningioma.^34^

It has been reported that depression is common prior to surgery but declines again following surgery to a level comparable to the general population.^20^ However, this observation was not reproduced at group level in our study where there was a consistent increase in use of antidepressants postoperatively compared to matched controls. Thus, our findings are in line with a cross sectional study, demonstrating that depressive symptoms and anxiety were common in patients with meningioma at an average of 33 months postoperatively.^21^ Interestingly, 43% of patients with preoperative antidepressant use did not require antidepressant treatment two years after surgery, i.e. considerably more than controls for whom this rate was 28% (*p* < 0.01).

### 4.3 Sedatives

Sedatives can provide effective short‐term symptom relief, but long‐term use is problematic. Benzodiazepine use exceeding 1–2 weeks increase the risk for dependency^35^ and falls.^36^ It is therefore discouraged.

The baseline level of 5%–7% use of sedatives among both patients and controls two years before surgery is comparable to previous reports.^37^ Sedative use for patients peaked around the time of surgery with 14.4% versus 6.1% for controls (*p* < 0.01). This may have been caused by anxiety and sleep disturbances following meningioma surgery. A similar pattern was shown in a Norwegian study from 2016 on prescriptions after total hip arthroplasty.^38^ More worrisome given the side effects of this class of drugs is that there is also an increased use two years after surgery among patients with 9.9% vs 6.9% for controls (*p* < 0.01). In the multivariable regression model, use of sedatives at the time of surgery was a strong predictor of continued use 2 years after surgery (*p* < 0.01). It is worth noting that higher age, while not being a predictor for continued AED or antidepressant use, was a predictor for continued use of sedatives. Hence, we recommend keeping the prescriptions of sedatives at a minimum, especially for elderly patients.

### 4.4 Clinical implications

Our findings can guide clinicians by shedding light on common problems for meningioma patients around the time of surgery. Increased awareness can aid in developing screening strategies to effectively identify issues and address them. The search for predictors may add further knowledge, in tailoring the surveillance to particular risk‐groups. Also, particularly for oligosymptomatic patients with limited tumor burden, knowledge of how softer end‐points are likely to develop after surgery may be of interest when considering the timing of surgical intervention.

### 4.5 Limitations of this study

When comparing large groups of drugs together, differences and nuances in prescription are unavoidably lost. It is difficult to interpret what a drug prescription means in terms of morbidity, and even harder to make causal conclusions. Therefore, in an effort to address this, we decided during study planning to exclude the drugs N03AX12 (Gabapentin) and N03AX16 (Pregabalin) from the AED group, as they are mainly used for the indication of pain relief in Sweden. Our interpretation of the results is that the main indication for antidepressants in this population was depressive symptoms, and that the main indication for sedatives was anxiety or sleep disturbances, but we have not measured the mood, anxiety level or quality of sleep of the patients directly.

Our method requires patients to purchase the drug in order to be counted as active users. A patient that receives AED in a hospital setting would be counted as a non‐user until this patient has purchased the AED in a store. This may have led to an underreporting of AED use in the weeks after surgery, but it is highly unlikely to have caused underreporting in patients with longer‐term AED use. It may also be considered a strength of the study that we used actual purchase, and not prescriptions, as a measure of drug use, since bias from non‐compliance is considerably lower when patients have purchased the drug. Indeed, the purchase of a prescribed drug is considered the definition of compliance in many studies.^39^

Disease registers have limited resolution over time and often lack details concerning risk factors when trying to explain findings or specific patterns. Therefore, we have chosen a descriptive pattern to avoid excessive speculation. We have studied only patients undergoing surgical resection of meningioma. Hence, we cannot compare against other modalities (e.g. radiotherapy) or simply conservative management as case selection clearly plays an important role.

### 4.6 Strengths of this study

Compared to other studies on the subject the 2070 patients included in this study is a very large cohort of surgically treated meningioma patients with both drug‐use data and high‐quality surgical data. We have used matched controls to compare our results to a baseline level in the general population. As we have studied use of drugs rather than prescriptions, bias from non‐adherence has been reduced. By organizing the drug use data using exact dates on an individual basis, we have achieved a time‐resolution uncommon for this type of study.

## 5 CONCLUSION

This nationwide study shows that use of antidepressants, AED, and sedatives was comparable between meningioma patients and controls two years before surgery but was increased for meningioma patients perioperatively and remained elevated during the entire studied period of two years after surgery.

## CONFLICT OF INTERESTS

None declared.

## ETHICS STATEMENT

This study was approved by the regional ethical committee in Västra Götaland region (Dnr: 363–17). The need for informed consent was waived by the ethical committee.

## Supporting information

Table S1‐S3

## Data Availability

Due to restrictions from the registry holders, raw data cannot be shared.
