# Supplementary material for: Impact of meningioma surgery on use of antiepileptic, antidepressant, and sedative drugs: A Swedish nationwide matched cohort study
Source: Cancer Med. 2021 Mar 26;10(9):2967–77. doi: 10.1002/cam4.3868 (PMC8085957; doi:10.1002/cam4.3868)
Supplement: Supplementary file 1 — Table S1‐S3 [file CAM4-10-2967-s001.docx]

**Supplementary table 1:** Multivariable logistic regression model for AED use at two years after meningioma surgery.

| **Variable** | **OR (95% CI)** | **P value** |
| --- | --- | --- |
| Index year | 0.92 (0.84-1.01) | 0.08 |
| AED-use at index date | 10.53 (7.70-14.39) | <0.01* |
| Female (vs male) | 0.91 (0.67-1.26) | 0.58 |
| Age (per year) | 1.00 (0.98-1.01) | 0.53 |
| Higher education (vs basic to high-school) | 0.60 (0.43-0.85) | <0.01* |
| Income (per 100 000 SEK) | 0.98 (0.90-1.07) | 0.67 |
| No symptoms preoperatively | 1.12 (0.68-1.84) | 0.65 |
| Elixhauser comorbidity index (0, 1, 2, ≥3) | 1.25 (1.07-1.45) | <0.01* |
| Atypical tumor (WHO grade II-III) | 1.01 (0.66-1.55) | 0.97 |
| Reoperation due to complication within 30 days | 0.62 (0.30-1.29) | 0.20 |
| New neurological deficit postoperatively | 2.04 (1.39-3.00) | <0.01* |
| Tumor size | 1.39 (1.11-1.72) | <0.01* |
| Skull-base location | 0.69 (0.44-1.07) | 0.10 |
| Functional level (per WHO category) | 1.26 (1.08-1.47) | <0.01* |
| Postoperative radiation treatment planned | 1.81 (1.00-3.28) | 0.05* |
| Wait time (per increasing quartile) | 0.86 (0.73-1.00) | 0.05* |

**Supplementary table 2:** Multivariable logistic regression model for antidepressant use at two years after meningioma surgery.

| **Variable** | **OR (95% CI)** | **P value** |
| --- | --- | --- |
| Index year | 0.91 (0.83-1.01) | 0.07 |
| Antidepressant use at index date | 11.89 (8.32-17.00) | <0.01* |
| Female (vs male) | 1.37 (0.92-2.03) | 0.12 |
| Age (per year) | 1.00 (0.99-1.01) | 0.88 |
| Higher education (vs basic to high-school) | 0.81 (0.56-1.18) | 0.27 |
| Income (per 100 000 SEK) | 0.98 (0.87-1.11) | 0.76 |
| No symptoms preoperatively | 1.10 (0.69-1.76) | 0.69 |
| Elixhauser comorbidity index (0, 1, 2, ≥3) | 0.99 (0.84-1.17) | 0.94 |
| Meningioma WHO grade II-III | 0.73 (0.41-1.28) | 0.27 |
| Reoperation due to complication within 30 days | 0.97 (0.42-2.23) | 0.94 |
| New neurological deficit postoperatively | 1.24 (0.78-1.97) | 0.36 |
| Tumor size | 0.86 (0.67-1.11) | 0.26 |
| Skull-base location | 1.00 (0.65-1.55) | 0.99 |
| Functional level (per WHO category) | 1.56 (1.31-1.85) | <0.01* |
| Postoperative radiation treatment planned | 1.01 (0.48-2.13) | 0.97 |
| Wait time (per increasing quartile) | 1.11 (0.94-1.31) | 0.20 |

**Supplementary table 3:** Multivariable logistic regression model for sedative use at two years after meningioma surgery.

| **Variable** | **OR (95% CI)** | **P value** |
| --- | --- | --- |
| Index year | 1.03 (0.92-1.15) | 0.61 |
| Sedative use at index date | 9.26 (6.27-13.70) | <0.01* |
| Female (vs male) | 1.04 (0.68-1.60) | 0.83 |
| Age (per year) | 1.02 (1.00-1.31) | 0.05* |
| Higher education (vs basic to high-school) | 1.22 (0.80-1. 84) | 0.35 |
| Income (per 100 000 SEK) | 0.92 (0.79-1.08) | 0.32 |
| No symptoms preoperatively | 1.59 (0.93-2.71) | 0.09 |
| Elixhauser comorbidity index (0, 1, 2, ≥3) | 1.38 (1.16-1.65) | <0.01* |
| Meningioma WHO grade II-III | 1.49 (0.85-2.62) | 0.16 |
| Reoperation due to complication within 30 days | 1.72 (0.77-3.83) | 0.19 |
| New neurological deficit postoperatively | 2.02 (1.24-3.28) | <0.01* |
| Tumor size | 0.77 (0.58-1.03) | 0.08 |
| Skull-base location | 0.73 (0.42-1.29) | 0.28 |
| Functional level (per WHO category) | 1.30 (1.07-1.58) | <0.01* |
| Postoperative radiation treatment planned | 0.69 (0.31-1.58) | 0.38 |
| Wait time (per increasing quartile) | 1.02 (0.85-1.24) | 0.81 |
